# Comparative Analysis of Bacterial Diversity and Community Structure in the Rhizosphere and Root Endosphere of Two Halophytes, *Salicornia europaea* and *Glaux maritima*, Collected from Two Brackish Lakes in Japan

**DOI:** 10.1264/jsme2.ME20072

**Published:** 2020-09-04

**Authors:** Kosuke Yamamoto, Minenosuke Matsutani, Yuh Shiwa, Taichiro Ishige, Hikaru Sakamoto, Hiromasa Saitoh, Seiya Tsushima

**Affiliations:** 1 Department of Molecular Microbiology, Faculty of Life Sciences, Tokyo University of Agriculture, Tokyo, Japan; 2 NODAI Genome Research Center, Tokyo University of Agriculture, Tokyo, Japan; 3 Department of Northern Biosphere Agriculture, Faculty of Bioindustry, Tokyo University of Agriculture, Hokkaido, Japan

**Keywords:** bacterial diversity, halophyte, rhizosphere bacteria, 16S rDNA, metagenome

## Abstract

Microbial community structures associated with halophytes and their compositions among different habitats, particularly natural saline sites, have not yet been investigated in detail. In the present study, we examined the diversity and composition of the rhizosphere and root endosphere bacteria of two halophytes, *Salicornia europaea* L. and *Glaux maritima* L., collected from two adjacent brackish lakes, Lake Notoro and Lake Tofutsu, in Japan. The bacterial species richness and diversity indices of the two halophytes collected from both lakes showed no significant differences in the rhizosphere or root endosphere. In contrast, beta diversity and taxonomic analyses revealed significant differences in the bacterial communities from each halophyte between the two lakes even though the two locations were natural saline sites, indicating that the bacterial communities for *S. europaea* and *G. maritima* both fluctuated in a manner that depended on the geographical location. Common and abundant genera associated with each halophyte across the two lakes were then identified to verify the bacterial genera specifically inhabiting each plant species. The results obtained showed that the composition of abundant genera inhabiting each halophyte across two lakes was distinct from that reported previously in other saline soil areas. These results suggest that each halophyte in different geographical sites had an individual complex bacterial community.

Soil salinization is one of the most critical environmental factors limiting the productivity of agricultural crops. According to the Food and Agriculture Organization (FAO) guidelines on sustainable food and agriculture for achieving the 2030 Agenda for Sustainable Development Goals (SDGs), salinization is one of the ten major threats to soil erosion because salts, particularly sodium salts, facilitate the diffusion of clay minerals, degrade the soil structure, and suppress water infiltration. The Voluntary Guidelines for Sustainable Soil Management from the FAO aim to prevent or minimize soil salinization, sodification, and alkalization (FAO, 2017, 2018a). The handbook for saline soil management from the FAO describes the utilization of halophytes as desalinization agents and phytoremediators for the reclamation of salt-affected soil. Halophytic plants uptake salts from soils and irrigation water and concentrate these salts in the above-ground biomass; halophytic plants with an above-ground biomass of 18–20 t ha^–1^ uptake salts from soil at a rate of 8–10 t ha^–1^ year^–1^ (FAO, 2018b). Therefore, salt-affected fields may be regenerated by the removal of above-ground plant parts.

Halophytes adapt well to high salinity conditions by using two strategies, salt tolerance and avoidance (Mishra and Tanna, 2017). Salt tolerance mechanisms include reductions in the influx of Na^+^, compartmentalization, and the excretion of sodium ions (Flowers and Colmer, 2015). Salt avoidance is a morphological response that functions through the secretion of excess salt and succulence of above-ground plant parts (Shabala, 2013; Shabala *et al.*, 2014). Salt-secreting structures, such as salt hairs or glands, are well-distributed in halophytes and have the capacity to excrete excess salt solution within halophytes.

In addition to the unique strategies used by halophytes, the root-associated microbial community plays an important role in adapting halophytic plants to high salt conditions (Redman *et al.*, 2002; Yuan *et al.*, 2016). Halotolerant plant growth-promoting rhizobacteria (PGPR) associated with halophytes enhance the growth and salt tolerance of their host plants. Rhizosphere bacteria from *Salicornia strobilacea* have been shown to stably colonize the rhizoplane and improve the growth of its host plant (Marasco *et al.*, 2016). Furthermore, the salt tolerance of *Arthrocnemum macrostachyum* was enhanced by its endophytic bacteria (Navarro-Torre *et al.*, 2017; Tian and Zhang, 2017). Increases in the above-ground biomass of halophytes are attributed to the ability of halotolerant PGPR to absorb salt more effectively from salt-affected soil; therefore, the combined use of halophytes and halotolerant PGPR may be useful in the reclamation of salt-damaged fields. However, the diversity of halotolerant PGPR in saline soil and halophyte-associated endophytic bacteria depends on the physicochemical properties of soil as well as the plant species (Qin *et al.*, 2016; Szymańska *et al.*, 2016). Therefore, interactions between certain halotolerant PGPR and halophytes may also depend on soil properties and/or the plant species. To address this issue, it is important to investigate the diversity of bacterial communities associated with the rhizosphere and root endosphere of various halophytes at different locations (Etesami and Beattie, 2018), which will clarify and describe ecological analogies between natural habitats of halophytes and salt-affected fields. To date, several studies have characterized the composition of the bacterial microbiota in the rhizosphere and root endosphere associated with the following halophytes: *Glaux maritima* (Yamamoto *et al.*, 2018), *Limonium sinense* (Qin *et al.*, 2018), *Messerschmidia sibirica* (Tian and Zhang, 2017), *Salicornia europaea* (Zhao *et al.*, 2016; Yamamoto *et al.*, 2018), and *Suaeda salsa* (Yuan *et al.*, 2016). We previously reported differences in bacterial diversity and community structure between *S. europaea* and *G. maritima* in an identical lake habitat (Yamamoto *et al.*, 2018). These findings prompted us to examine whether the bacterial diversity and community structure associated with each halophyte is distinct between two natural saline sites. Different bacterial communities in the shoots and roots of *S. europaea* were previously observed in a naturally saline site at which brine appeared from Zechstein salt deposits as well as in an anthropogenic pond with salinity due to soda factory wastewater; these findings demonstrated that differences in the origin of salinity at the two sites had an impact on the bacterial microbiome composition of *S. europaea* (Szymańska *et al.*, 2018; Furtado *et al.*, 2019). A previous study reported that the bacterial communities associated with the stems and roots of *A. macrostachyum* markedly differed between a natural location (salt marsh) and artificial location (sea salt farm), which was caused by differences in the soil saline composition of the two sites (Mora-Ruiz *et al.*, 2016). However, the bacterial communities associated with halophytes from two natural saline sites have not yet been simultaneously investigated using culture-independent methods, such as 16S rRNA gene sequencing.

Therefore, we herein examined the bacterial diversity and community structure of the rhizosphere and root endosphere of two halophytes, *S. europaea* L. and *G. maritima* L., collected from two brackish lakes, Lake Notoro and Lake Tofutsu, Japan. These lakes are located only 17.5‍ ‍km from each other and have the same origin of salinity, *i.e.*, the Sea of Okhotsk. Furthermore, to elucidate the bacterial genera associated with each halophyte, we identified common and abundant genera in the rhizosphere and root endosphere of each halophyte from the two lakes. The results of the present study will expand our understanding of the bacterial ecology of the rhizosphere and root endosphere in halophytes.

## Materials and Methods

### Sample collection and fractionation

**Fig. 1.**
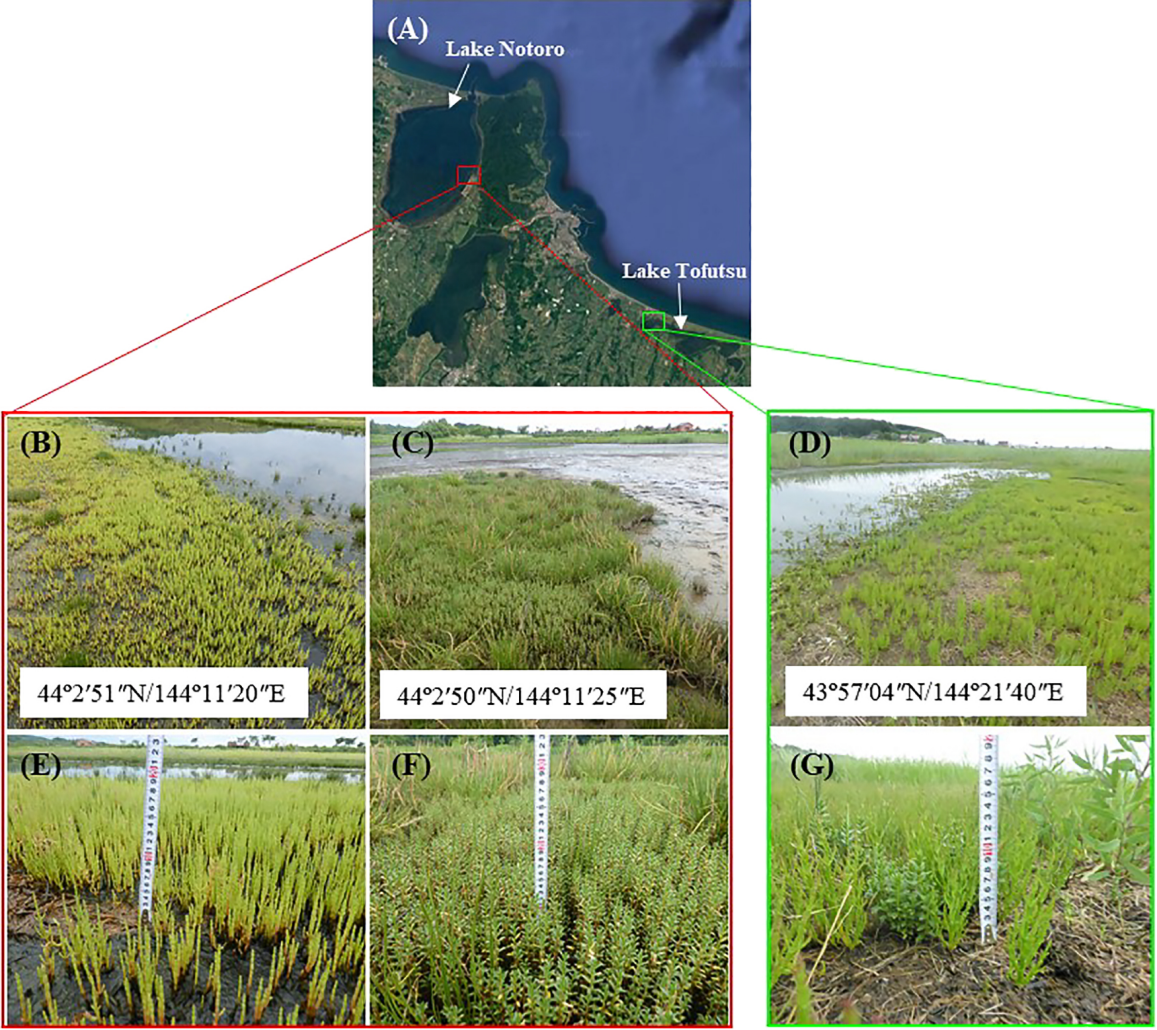
Study area. Each sample was collected from both ‘Lake Notoro’ and ‘Lake Tofutsu’ (eastern part of Hokkaido, Japan). Satellite imagery was obtained using Google earth pro software (A). *Salicornia europaea* (B and E) and *Glaux maritima* (C and F) grew at two different tidal areas in Lake Notoro. In contrast to Lake Notoro, the two halophytes (D and G) grew at the same location in Lake Tofutsu. Each halophyte was harvested and the root system was sampled as described in the *Materials and Methods*.

We sampled both *S. europaea* and *G. maritima* from Lake Notoro and Lake Tofutsu in the eastern part of Hokkaido, Japan, in July 2017 at low tide. Since the two halophytes constituted individual communities in Lake Notoro, *S. europaea* was collected from 44°2′51″N/144°11′20″E and *G. maritima* from 44°2′50″N/144°11′25″E as described previously (Yamamoto *et al.*, 2018). On the same date, the two halophytes were also collected from Lake Tofutsu (Fig. 1). In contrast to Lake Notoro, the two halophytes grew at the same location (43°57′04″N/144°21′40″E) in Lake Tofutsu. Sampling from both lakes was conducted as permitted by Hokkaido Prefecture. Sampling was performed according to the method described by Schlaeppi *et al.* (2014). We excavated whole plants including the surrounding soil in blocks (~20‍ ‍cm in length, ~20‍ ‍cm in width, and 10–20‍ ‍cm in depth). The plants in their soil cores were brought to the laboratory, and the root systems were sampled within 12 h of the removal of the plants from their natural habitat. Twenty individual plants were pooled into four samples: each sample contained five plants. The rhizosphere and root compartments from four samples per species were fractionated according to previously described methods (Schlaeppi *et al.*, 2014; D’Amico *et al.*, 2018). Root segments were cut into pieces that were 3‍ ‍cm in length, starting 0.5‍ ‍cm below the root base. These root segments were placed into 15-mL sterile tubes containing 10‍ ‍mL PBS-S buffer (130‍ ‍mM NaCl, 7‍ ‍mM Na_2_HPO_4_, 3‍ ‍mM NaH_2_PO_4_, pH 7.0, and 0.02% Silwet L-77) and shaken at 180 rpm for 20‍ ‍min for washing. The roots were then transferred to a new 15-mL sterile tube, and the remaining soil suspension was centrifuged at 4,000×*g* for 20‍ ‍min. The pellet was defined as the rhizosphere (Rh) sample and frozen in liquid nitrogen for storage at –‍80°C. After a second wash under the same conditions, the roots were transferred to a new 15-mL sterile tube with 10‍ ‍mL PBS-S buffer and subjected to sonication for 10‍ ‍min with a water bath sonicator at 40 kHz (Model 5510; Branson Ultrasonics). Following an additional wash using the same procedure described above, root samples were transferred to a new 15-mL sterile tube and then frozen in liquid nitrogen for storage at –80°C. These root samples were defined as root endosphere (Re) samples. After all the plants had been harvested from the soil block, bulk soil samples were collected from 0.5 to 3.5‍ ‍cm of the soil surface, which corresponded to the same area as the root sample, frozen in liquid nitrogen, and stored at –‍80°C. The Rh, Re, and bulk control soil (Bl) samples collected were used for DNA extraction.

### DNA extraction, PCR amplification, and sequencing

Regarding Rh and Bl, 0.5‍ ‍g of each sample was used for DNA extraction. Re samples were frozen in liquid nitrogen and ground into a fine powder with a sterilized mortar and pestle, and 0.5‍ ‍g of the powder was used for DNA extraction. Total DNA was extracted using a NucleoSpin Soil Kit (Macherey-Nagel) containing buffer SL1 and enhancer SX, which have previously been used to extract high-quality DNA in large quantities from paddy soil (Knauth *et al.*, 2013).

The bacterial amplicon library was generated using a primer set targeting the V3–V4 hypervariable region of the 16S rRNA gene as described previously (Yamamoto *et al.*, 2018). The libraries were sequenced as paired-ends and 300-bp reads on an Illumina MiSeq sequencer (run center: the NODAI Genome Research Center). All sequenced data obtained in the present study have been deposited in the DDBJ Sequence Read Archive (DRA) database under the accession numbers DRA006852 and DRA009815.

### Sequence processing and analysis

Sequence processing was performed according to the method described by Zabat *et al.* (2018). Raw paired-end FASTQ files were quality-filtered, trimmed, de-noised, and merged using DADA2 (Callahan *et al.*, 2016) in QIIME2 (ver. 2017.11, https://qiime2.org/). After the chimeric sequences were removed, the consensus method in DADA2 was used to cluster the sequences into operational taxonomic units (OTUs) at 100% identity. A taxonomic analysis of OTUs was performed using the QIIME2 q2-feature-classifier plugin with a pre-trained Naïve Bayes classifier on the SILVA 99% OTU database (version 128; http://www.arb-silva.de) trimmed to the V3-V4 region of the 16S rRNA gene (DeSantis *et al.*, 2006). Multiple sequence alignments and phylogenetic reconstructions were conducted using MAFFT and FastTree, respectively (Price *et al.*, 2009; Katoh and Standley, 2013).

### Statistical analysis

Subsequent analyses were performed using R ver. 3.5.2 with the phyloseq (McMurdie and Holmes, 2013) and pheatmap (Kolde, 2015) packages. OTUs classified as chloroplasts and mitochondria were filtered out. Alpha diversity was estimated using the Shannon and Chao1 indices with an absolute abundance matrix. The relationships between the bacterial community structures from the two halophytes were evaluated using a principal coordinate analysis (PCoA) and complete linkage clustering (CLC) based on weighted UniFrac distances from the relative abundance matrix and were statistically confirmed using PERMANOVA.

## Results

### Analyses of 16S rRNA gene sequencing data

In total, 27,232,388 raw reads were obtained from the Miseq sequencing analysis of 44 samples, ranging between 1,284,467 and 334,380 reads in each sample. After read-quality filtering, 12,823,193 quality-filtered reads were obtained, ranging between 625,901 and 142,027 reads in each sample with an average length of 419–416 bp. A total of 116,830 OTUs were extracted, ranging between 5,040 and 1,074 OTUs in each sample (Table S1). As shown in Fig. S1, the rarefaction curve for the OTUs obtained from each individual sample was almost saturated, indicating that most of the bacterial species had been sampled.

### Species richness and diversity of bacteria associated with two halophytes collected from two brackish lakes

**Fig. 2.**
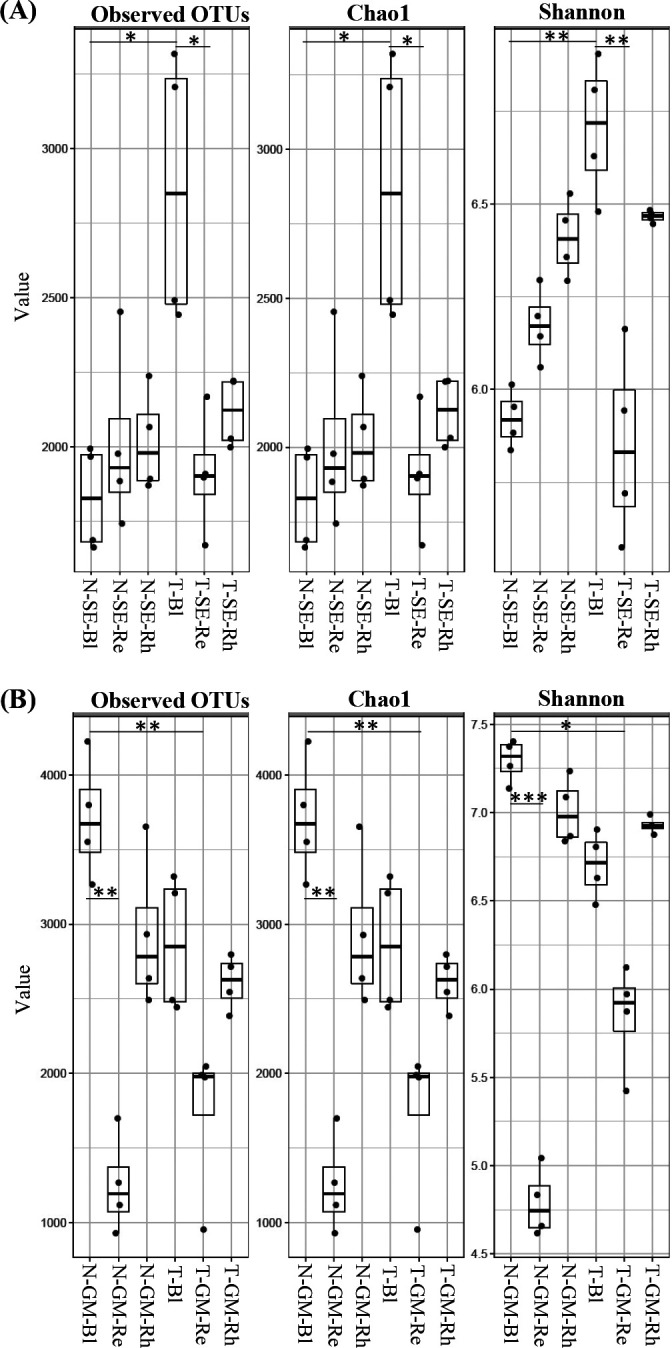
Alpha diversity indices for 16S rDNA sequences. Box plots show observed OTUs, Chao1, and Shannon indices in bulk control soil, rhizosphere, and root endosphere samples associated with *Salicornia europaea *(A) or *Glaux maritima* (B) collected from two brackish lakes, Lake Notoro and Lake Tofutsu. Whiskers represent minimum and maximum values. All other points are contained within the box, and the bar represents the median. A Holm-adjusted *P*-value was calculated from Dunn’s test of multiple comparisons using rank sums. Asterisks indicate significant differences between pairs (*P<0.05, **P<0.01 and ***P<0.001). Data related to *S. europaea* or *G. maritima* collected from Lake Notoro were used in a previous study (Yamamoto *et al.*, 2018). N, Lake Notoro; T, Lake Tofutsu; SE, *S. europaea*; GM, *G. maritima*; Bl, bulk control soil; Rh, rhizosphere; Re, root endosphere.

In *S. europaea* samples, the Bl sample from Lake Tofutsu (T-Bl) had the highest bacterial species richness and diversity, and exhibited significantly higher OTUs (*P*=0.0122 against N-SE-Bl and *P*=0.0485 against T-SE-Re), Chao1 (*P*=0.0122 against N-SE-Bl and *P*=0.0485 against T-SE-Re), and Shannon (*P*=0.0047 against N-SE-Bl and *P*=0.0035 against T-SE-Re) indices than those of the Bl sample from Lake Notoro (N-SE-Bl) and Re sample from Lake Tofutsu (T-SE-Re) (Fig. 2A and Table 1). No significant differences were observed in alpha diversity between the Rh and Re samples from Lake Notoro (N-SE-Rh and N-SE-Re) and Lake Tofutsu (T-SE-Rh and T-SE-Re). On the other hand, comparisons of the alpha diversity metrics of *G. maritima* samples from both lakes revealed that the two Re samples (N-GM-Re and T-GM-Re) had significantly lower OTUs (*P*=0.0016 and *P*=0.0096), Chao1 (*P*=0.0016 and *P*=0.0096), and Shannon (*P*=0.0006 and *P*=0.0114) indices, respectively, than those of the Bl sample from Lake Notoro (N-GM-Bl) (Fig. 2B and Table 1). Similar to the alpha diversity of the *S. europaea* Rh and Re samples, no significant differences were observed in alpha diversity between *G. maritima* Rh and Re samples from Lake Notoro (N-GM-Rh and N-GM-Re) and Lake Tofutsu (T-GM-Rh and T-GM-Re).

### Comparative analysis of bacterial communities associated with halophytes collected from two brackish lakes

**Fig. 3.**
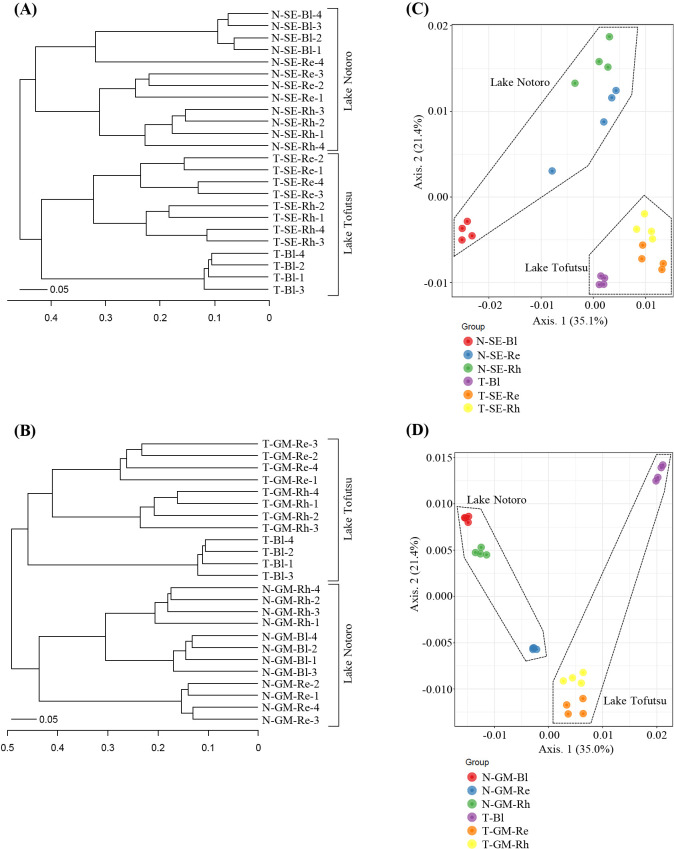
Complete linkage clustering (CLC) (A and B) and principal coordinate analysis (PCoA) (C and D) of bacterial communities in different samples associated with *Salicornia europaea* or *Glaux maritima* collected from two brackish lakes based on weighted UniFrac distances. Data related to *S. europaea* or *G. maritima* collected from Lake Notoro were used in a previous study (Yamamoto *et al.*, 2018). N, Lake Notoro; T, Lake Tofutsu; SE, *S. europaea*; GM, *G. maritima*; Bl, bulk control soil; Rh, rhizosphere; Re, root endosphere.

To compare the bacterial community structure associated with each halophyte collected from Lake Notoro and Lake Tofutsu, a beta diversity analysis was performed based on CLC (Fig. 3A and B) and PCoA (Fig. 3C and D). In the CLC tree, all samples were clustered into two groups according to habitat. Four replicates of the Bl, Rh, and Re samples for each halophyte clustered together, except for the *S. europaea* Re sample from Lake Notoro (Fig. 3A and B). Similar results were obtained from PCoA. Axis 1 showed the influence of habitat, which explained 35.1 and 35.0% variations for *S. europaea* (Fig. 3C) and *G. maritima* (Fig. 3D) samples, respectively. The quadruplicate Bl, Rh, and Re samples obtained for each halophyte also appeared to be clustered together, explaining 21.4% (axis 2) of the variation (Fig. 3C and D). These results indicated that the microbiota of the three sample types for each halophyte from Lake Notoro markedly differed from those from Lake Tofutsu.

### Bacterial taxonomic analysis at the phylum level

**Fig. 4.**
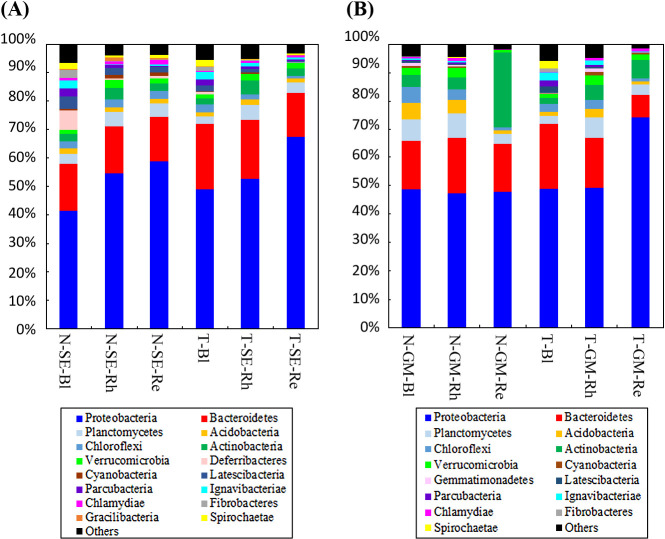
Average relative abundance of bacteria at the phylum level in different samples associated with* Salicornia europaea* (A) or *Glaux maritima* (B) collected from two brackish lakes. Data related to *S. europaea* or *G. maritima* collected from Lake Notoro were used in a previous study (Yamamoto *et al.*, 2018). N, Lake Notoro; T, Lake Tofutsu; SE, *S. europaea*; GM, *G. maritima*; Bl, bulk control soil; Rh, rhizosphere; Re, root endosphere.

In the *S. europaea* samples from both lakes, 58 phyla were identified (Table S2). The relative abundance of the top 16 phyla (>1% of relative abundance in at least one sample) is shown in Fig. 4A. These results represent the average of four replicates. *Proteobacteria* and *Bacteroidetes* were the dominant phyla (>10% of relative abundance) across all samples, accounting for 41.5–67.5 and 15.3–23.0%, respectively, of all high-quality sequences. *Planctomycetes*, *Acidobacteria*, *Chloroflexi*, *Actinobacteria*, and *Verrucomicrobia* were the sub-dominant phyla (>1% of relative abundance) in all samples, accounting for 2.6–5.3, 1.2–1.9, 1.0–2.9, 2.0–4.7, and 1.4–2.8%, respectively, of all high-quality sequences. The relative abundance of *Cyanobacteria*, *Latescibacteria*, *Chlamydiae*, *Gracilibacteria*, and *Spirochaetae* was higher in the Rh and Re samples from Lake Notoro (N-SE-Rh and N-SE-Re) than in those from Lake Tofutsu. On the other hand, *Ignavibacteriae* showed more than 1% relative abundance in the Rh samples from Lake Tofutsu (T-SE-Rh), but not in the samples from Lake Notoro. *Deferribacteres*, *Parcubacteria*, and *Fibrobacteres* were the sub-dominant phyla in the Bl samples from Lake Notoro and Lake Tofutsu only (N-SE-Bl and T-Bl). The other 42 phyla had low abundance (less than 1% of high-quality sequences). Therefore, these phyla were defined as rare phyla and referred to as “others” in Fig. 4A.

In the *G. maritima* samples from the two lakes, 59 phyla were identified (Table S3). The relative abundance of the top 15 phyla (>1% of relative abundance in at least one sample) is shown in Fig. 4B. *Proteobacteria* was the dominant phyla (>10% of relative abundance) across all samples, accounting for 47.4–74.1% of all high-quality sequences. *Bacteroidetes*, *Actinobacteria*, *Planctomycetes*, and *Chloroflexi* were the sub-dominant phyla (>1% of relative abundance) in all samples, accounting for 8.1–23.0, 2.0–26.3, 2.6–8.6, and, 1.0–5.7%, respectively, of all high-quality sequences. *Verrucomicrobia* and *Acidobacteria* were the sub-dominant phyla (>1% of relative abundance) in most of the samples, except for the Re samples from both lakes. The relative abundance of *Cyanobacteria* and *Gemmatimonadetes* was higher in the Rh sample from Lake Tofutsu (T-GM-Rh) than in that from Lake Notoro. *Ignavibacteriae* was the sub-dominant phyla in the Bl and Rh samples from Lake Tofutsu (T-Bl and T-GM-Rh), but not in those from Lake Notoro. *Chlamydiae* was the sub-dominant phyla in the Rh samples from Lake Notoro (N-‍GM-Rh), but not in those from Lake Tofutsu. The relative abundance of *Latescibacteria*, *Parcubacteria*, *Fibrobacteres*, and *Spirochaetae* was more than 1% of the high-quality sequences identified in the Bl sample from Lake Tofutsu (T-Bl). The 44 low abundance (less than 1% of high-quality sequences) phyla were defined as rare phyla and referred to as “others” in Fig. 4B.

### Bacterial taxonomic analysis at the genus level

Comparisons of the relative abundance of the top 50 classified genera based on a heatmap analysis revealed the significant influence of habitat differences in the bacterial community structure among the sample types. Furthermore, more abundant genera, which were defined as having at least 2-fold higher relative abundance among the sample types, were distributed in each Rh and Re sample related to the two halophytes from the two lakes.

**Fig. 5.**
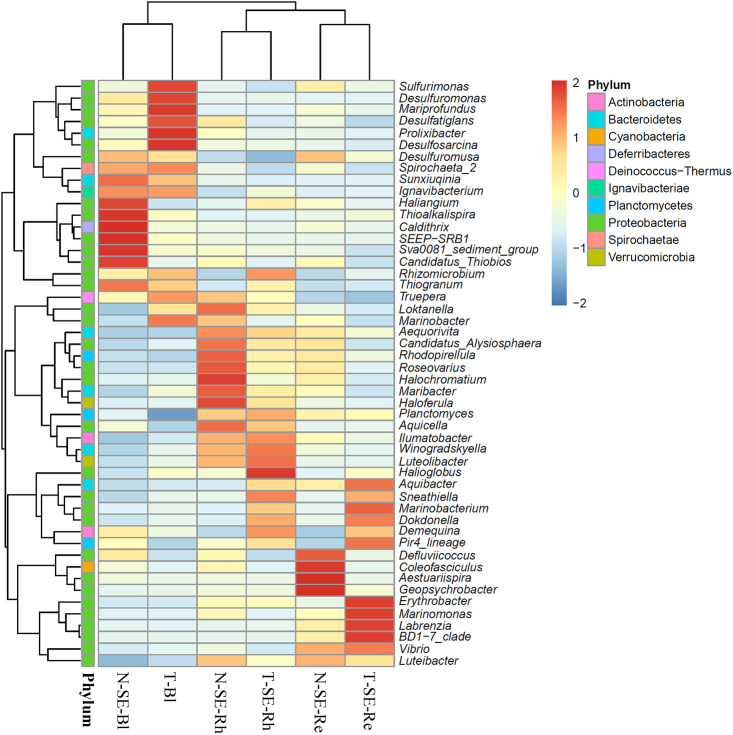
Heatmap and cluster analyses of the bacterial distribution of the top 50 abundant genera in three *Salicornia europaea* sample types collected from two brackish lakes. The dendrogram shows complete linkage agglomerative clustering based on the Euclidean distance. The heatmap color (blue to red) represents the row *z*-score of the mean relative abundance from low to high. Data related to *S. europaea* or *Glaux maritima* collected from Lake Notoro were used in a previous study (Yamamoto *et al.*, 2018). N, Lake Notoro; T, Lake Tofutsu; SE, *S. europaea*; Bl, bulk control soil; Rh, rhizosphere; Re, root endosphere.

Fig. 5 shows clustering of the top 50 classified genera in the *S. europaea* Bl, Rh, and Re samples from the two lakes. These genera belonged to 10 phyla: 32 genera belonged to *Proteobacteria*, 6 to *Bacteroidetes*, 3 to *Plactomycetes*, 2 to *Actinobacteria*, 2 to *Verrucomicrobia*, 1 to *Cyanobacteria*, 1 to *Deferribacteres*, 1 to *Deinococcus-Thermus*, 1 to *Ignavibacteriae*, and 1 to *Spirochaetae* (Fig. 5 and Table S4). The distribution of genera markedly differed across different samples. *Maribacter*, *Roseovarius*, *Halochromatium*, and *Haloferula* in the Rh sample from Lake Notoro (N-SE-Rh) had at least 2-fold higher relative abundance that that in other samples, whereas only the genus *Halioglobus* was more abundant in the Rh samples from Lake Tofutsu (T-SE-Rh). *Defluviicoccus*, *Coleofasciculus*, *Aestuariispira*, and *Geopsychrobacter* exhibited the highest abundance (>2-fold higher relative abundance) in the Re samples from Lake Notoro (N-SE-Re). *Erythrobacter*, *Marinomonas*, *Labrenzia*, and *BD1-7 clade* showed the highest relative abundance in the Re samples from Lake Tofutsu (T-SE-Re).

**Fig. 6.**
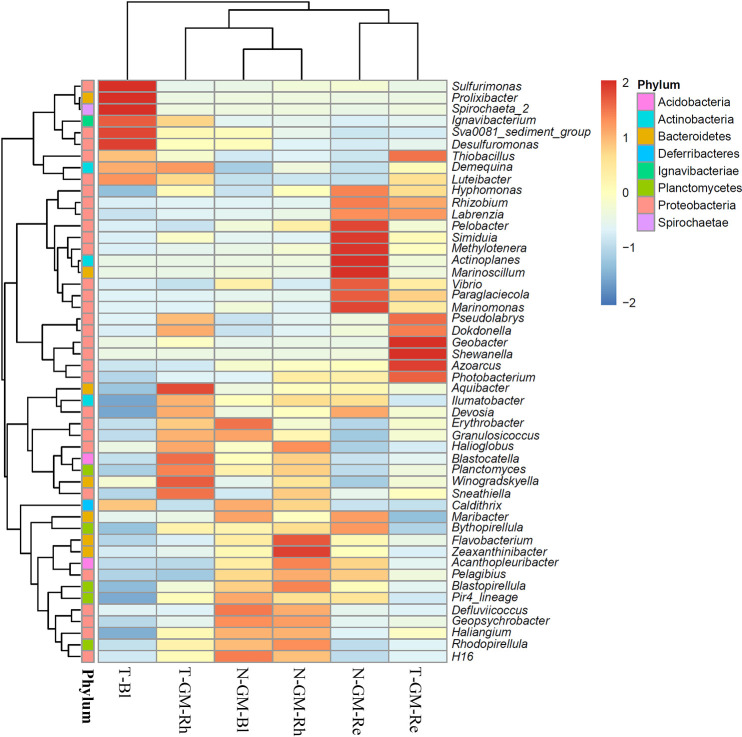
Heatmap and cluster analyses of the bacterial distribution of the top 50 abundant genera in three *Glaux maritima* sample types collected from two brackish lakes. The dendrogram shows complete linkage agglomerative clustering based on the Euclidean distance. The heatmap color (blue to red) represents the row *z*-score of the mean relative abundance from low to high. Data related to *Salicornia europaea* or *G. maritima* collected from Lake Notoro were used in a previous study (Yamamoto *et al.*, 2018). N, Lake Notoro; T, Lake Tofutsu; GM, G.* maritima*; Bl, bulk control soil; Rh, rhizosphere; Re, root endosphere.

Fig. 6 shows a heatmap of the top 50 classified genera in the *G. maritima* Bl, Rh, and Re samples from the two lakes. The top 50 classified genera belonged to 8 phyla: 30 genera belonged to *Proteobacteria*, 7 to *Bacteroidetes*, 5 to *Plactomycetes*, 3 to *Actinobacteria*, 2 to *Acidobacteria*, 1 to *Deferribacteres*, 1 to *Ignavibacteriae*, and 1 to *Spirochaetae* (Table S5). *Zeaxanthinibacter* showed the highest relative abundance (>2-fold higher relative abundance) in the Rh sample from Lake Notoro (N-GM-Rh); however, there was no significantly enriched genus in the Rh sample from Lake Tofutsu. *Pelobacter*, *Simiduia*, *Methylotenera*, *Actinoplanes*, *Marinoscillum*, and *Marinomonas* were the more abundant genera in the Re sample from Lake Notoro (N-GM-Re), whereas *Geobacter*, *Shewanella*, and *Azoarcus* were distributed more abundantly in the Re sample from Lake Tofutsu (T-SE-Re).

### Analysis of common and abundant bacteria at the genus level

An abundant genus was defined as a genus having >0.5% relative abundance in the top 50 classified genera in the Bl, Rh, and Re samples associated with each halophyte from Lake Notoro or Lake Tofutsu, and common and abundant genera across the two brackish lakes were then estimated in‍ ‍the Rh and Re samples related to each halophyte. As shown in Table 2, *Aquibacter*, *Luteibacter*, *Demequina*, *Planctomyces*, and *Winogradskyella* were the common and abundant genera in the *S. europaea* Rh and Re samples from both lakes. The common and abundant genera in *S. europaea* Rh samples only across the two lakes were *Ilumatobacter*, *Halochromatium*, *Candidatus Alysiosphaera*, *Sva0081 sediment group*, and *Pir4 lineage*. *Labrenzia*, *BD1-7 clade*, *Sulfurimonas*, and *Vibrio* were the common and abundant genera in the *S. europaea* Re samples across the two lakes (Table 2, S6, and S7).

On the other hand, the common and abundant genera in the *G. maritima* Rh and Re samples from the two lakes were *Aquibacter*, *Ilumatobacter*, and *Pir4 lineage* (Table 3, S8, and S9). The following eight genera, *Planctomyces*, *Winogradskyella*, *Blastocatella*, *Sneathiella*, *Demequina*, *Halioglobus*, *Rhodopirellula*, and *Haliangium* were common and abundant in the *G. maritima* Rh samples across the two lakes. Furthermore, the seven common and abundant genera in the *G. maritima* Re samples across the two lakes were as follows: *Paraglaciecola*, *Photobacterium*, *Marinomonas*, *Rhizobium*, *Labrenzia*, *Vibrio*, and *Actinoplanes* (Table 3, S8, and S9). The following eight abundant genera, *Aquibacter*, *Ilumatobacter*, *Pir4 lineage*, *Planctomyces*, *Winogradskyella*, *Demequina*, *Labrenzia*, and *Vibrio*, generally existed in the Rh and/or Re samples associated with both *S. europaea* and *G. maritima* across the two lakes.

## Discussion

The alpha diversity indices of the Rh and Re samples of *S. europaea* and *G. maritima* showed no significant differences between Lake Notoro and Lake Tofutsu. Szymańska *et al.* (2018) reported no significant differences in the diversity indices of the root-associated endophytic bacteria of *S. europaea* between natural or anthropogenic saline sites, which had significantly different soil parameters. Therefore, alpha diversity indices for the two halophytes may not be susceptible to location effects, suggesting a similarity in the microniches available for bacteria in Lake Notoro and Lake Tofutsu. On the other hand, based on beta diversity analyses using CLC and PCoA, the three sample types (Bl, Rh, and Re) related to each halophyte showed completely different bacterial community structures in the two natural saline sites, which were located only 17.5‍ ‍km from each other and had the same origin of salinity, the Sea of Okhotsk. These results were also supported by heatmap analyses at the genus level, *i.e.*, the rhizosphere and root endosphere associated with each halophyte retained unique “more abundant genera” in both natural lakes (Fig. 5 and 6). Consistent with the present results, Furtado *et al.* (2019) reported differences in bacterial community compositions in the roots and rhizosphere related to *S. europaea* at two test sites, *i.e.*, natural and anthropogenic saline sites. Therefore, the present results and previous findings collectively indicate that the bacterial community structures for both *S. europaea* and *G. maritima* fluctuated in a manner that depended on the geographical location.

In all samples of the two halophytes, the most dominant bacterial phyla were *Proteobacteria* followed by *Bacteroidetes*, *Planctomycetes*, *Chloroflexi*, and *Actinobacteria* (>1% of high-quality sequences in all tested samples). Previous studies also demonstrated that *Proteobacteria* was the most represented phylum, followed by *Bacteroidetes* in the root endosphere associated with *S. europaea* (Zhao *et al.*, 2016; Szymańska *et al.*, 2018; Furtado *et al.*, 2019). Therefore, bacteria classified into the phyla *Proteobacteria* and *Bacteroidetes* may play important roles in the environmental ecology of both *S. europaea* and *G. maritima* because these phyla are dominant not only in *S. europaea*, but also in other halophytes (Shi *et al.*, 2015; Mukhtar *et al.*, 2017; Tian and Zhang, 2017). Szymańska *et al.* (2018) recently demonstrated that *Planctomycetes*, *Chloroflexi*, and *Actinobacteria* were the dominant phyla among the root endophytes for *S. europaea* across the tested sites, which is consistent with the present results.

We speculated that the common and abundant genera (>0.5% of relative abundance; He *et al.*, 2017; Karimi *et al.*, 2018) present across the two brackish lakes were specific bacterial genera associated with each halophytic plant species; therefore, we defined the common and abundant genera related to the Rh and Re samples within the top 50 classified genera in the three sample types (Bl, Rh, and Re) related to each halophyte from each brackish lake. In the *S. europaea* Rh and Re samples, 14 genera were identified as common and abundant genera (Table 2). Within these genera, *Vibrio* was previously described as an abundant genus in the roots of *S. europaea* (Furtado *et al.*, 2019), whereas the other bacterial genera were only abundant in the present study. Similarly, 18 common and abundant genera were distinguished in the *G. maritima* Rh and Re samples (Table 3). The following four genera, *Haliangium*, *Rhizobium*, *Actinoplanes*, and *Vibrio* were previously reported as abundant genera associated with one of the following three halophytes: *A. macrostachyum*, *M. sibirica*, and *S. europaea*. (Zhao *et al.*, 2016; Tian and Zhang, 2017; Camacho-Sanchez *et al.*, 2020). However, the 14 other genera were identified as abundant genera in the present study only. These results indicate that the composition of abundant genera inhabiting each halophyte across two lakes is distinct from that in other saline soil areas reported previously, and the diversity of genera inhabiting these areas may also depend on the geographical location.

We also attempted to identify potential halotolerant PGPR candidates within the common and abundant genera, which have been reported to have PGP traits. Tables 2 and 3 summarize the possible functions of these common and abundant bacterial genera. *Luteibacter* was the common and abundant genus in the root endosphere and rhizosphere of *S. europaea*. *Luteibacter* functions in the chelation of ferric ions, solubilization of monocalcium phosphate, and production of indole-3-acetic acid (IAA) (Guglielmetti *et al.*, 2013), suggesting a role in the rhizosphere and root endosphere of *S. europaea* (Table 2). In addition, the sulfur-oxidizing bacterial genus, *Sulfurimonas*, was common and abundant in the root endosphere in *S. europaea*. A previous study indicated functions for *Sulfurimonas* in host detoxification by oxidizing sulfide and producing sulfate as an end-product, suggesting that the accumulation of these bacteria around the rhizosphere is important for the host tolerance of coastal environments (Fahimipour *et al.*, 2017). Furthermore, the genus *Vibrio* was common and abundant in the root endosphere of *S. europaea*. Gutierrez *et al.* (2009) reported that *Vibrio* spp. isolated from the roots of estuarine grasses produced IAA. Therefore, the genera *Luteibacter*, *Sulfurimonas*, and *Vibrio* may play important roles in the growth of *S. europaea* in coastal areas; however, it currently remains unclear whether these genera exert beneficial effects on plant growth in salt-affected fields. On the other hand, the common and abundant genera in the root endosphere of *G. maritima* were *Marinomonas*, *Rhizobium*, and *Actinoplanes*. Many species of *Marinomonas*, *Rhizobium*, and *Actinoplanes* have been reported to exert plant growth-promoting effects on host plants, such as the production of IAA, indole-3-pyruvic acid (IPYA), and gibberellic acid (GA_3_), antifungal activity, N_2_ fixation, phosphate solubilization, siderophore production, and 1-aminocyclopropane-1-carboxylate (ACC) deaminase activity (Table 3; El-Tarabily *et al.*, 2009; Jha *et al.*, 2012; Mesa *et al.*, 2015; Sorty *et al.*, 2016). Furthermore, the plant growth-promoting abilities of several *Rhizobium* spp. have been demonstrated in the following three halophytes: *Psoralea corylifolia*, *S. brachiate*, and *S. bigelovii* (Etesami and Beattie, 2018). In addition, the following three genera, *Haliangium*, *Photobacterium*, and *Vibrio*, were common and abundant in the rhizosphere of *G. maritima* (Table 3). The genus *Haliangium* includes species that produce haliangicin, an antifungal metabolite (Fudou *et al.*, 2001). Mathew *et al.* (2015) reported that *Photobacterium* spp. strain MELD1 exhibited mercury-reducing and IAA-producing activities. Therefore, the six genera described above may exert beneficial effects on the host plant. Previous studies demonstrated that the inoculation of bacterial species (*R. leguminosarum*) belonging to the genus *Rhizobium* promoted root and shoot growth in maize, lettuce, and wheat under greenhouse and field conditions (Mehboob *et al.*, 2009), indicating the benefits of the combined use of *Rhizobium* inhabiting *G. maritima* and halophytes in the regeneration of salt-damaged fields. However, the functions of any of the other bacteria in their host plants currently remain unclear (Table 2 and 3).

In conclusion, the present results revealed marked differences in bacterial components (beta diversity) between the same halophytes collected from two natural saline sites (Lake Notoro and Lake Tofutsu). We also demonstrated the distinct compositions of the abundant genera inhabiting each halophyte across two lakes from those in other saline soil areas reported previously. These results suggest that the bacterial community structures associated with halophytes in salt-damaged fields also depend on geographical locations.

Moreover, we observed differences in bacterial genera that have been reported to have PGP traits from *S. europaea* and *G. maritima* samples. Some common and abundant genera may promote the growth of each halophyte in the saline environment as halotolerant PGPR. Further studies are needed to clarify the function of the bacteria belonging to these genera as halotolerant PGPR and whether these bacteria play important roles in the interactions between plants and microbes in coastal areas.

## Citation

Yamamoto, K., Matsutani, M., Shiwa, Y., Ishige, T., Sakamoto, H., Saitoh, H., and Tsushima, S. (2020) Comparative Analysis of Bacterial Diversity and Community Structure in the Rhizosphere and Root Endosphere of Two Halophytes, *Salicornia europaea* and *Glaux maritima*, Collected from Two Brackish Lakes in Japan. *Microbes Environ ***35**: ME20072.

https://doi.org/10.1264/jsme2.ME20072

## Supplementary Material

Supplementary Material 1

Supplementary Material 2

Supplementary Material 3

Supplementary Material 2

Supplementary Material 2

Supplementary Material 2

Supplementary Material 2

Supplementary Material 2

Supplementary Material 2

Supplementary Material 2

## Figures and Tables

**Table 1.** Summary of bacterial richness and diversity indices of each *Salicornia europaea* and *Glaux maritima* sample collected from Lake Notoro and Lake Tofutsu (brackish lakes).

Sample name	Sample origin	Plant species	Observed OTUs	Chao1	Shannon
N-GM-Bl	Bulk control soil	*G. maritima*	3,710±405	3,710±405	7.29±0.12
N-GM-Re	Root endosphere	*G. maritima*	1,254±326	1,254±326	4.79±0.19
N-GM-Rh	Rhizosphere	*G. maritima*	2,928±515	2,929±515	7.01±0.19
T-Bl	Bulk control soil	*S. europaea* and *G. maritima*	2,866±461	2,866±462	6.70±0.19
T-GM-Re	Root endosphere	*G. maritima*	1,740±526	1,740±526	5.85±0.30
T-GM-Rh	Rhizosphere	*G. maritima*	2,610±183	2,610±184	6.93±0.05
N-SE-Bl	Bulk control soil	*S. europaea*	1,828±176	1,828±176	5.92±0.08
N-SE-Re	Root endosphere	*S. europaea*	2,015±308	2,015±308	6.17±0.1
N-SE-Rh	Rhizosphere	*S. europaea*	2,018±171	2,018±171	6.41±0.1
T-SE-Re	Root endosphere	*S. europaea*	1,912±203	1,912±203	5.85±0.26
T-SE-Rh	Rhizosphere	*S. europaea*	2,117±120	2,118±120	6.47±0.02

Data related to *S. europaea* or *G. maritima* collected from Lake Notoro were used in a previous study (Yamamoto *et al.*, 2018).

**Table 2.** Possible functions of common and abundant bacterial genera in the root endosphere and/or rhizosphere of *Salicornia europaea* across Lake Notoro and Lake Tofutsu.

Genus	Sample type	Possible function	References
*Aquibacter*	Rh, Re	Zeaxanthin-producing activity. There are no studies on beneficial effects on host plants.	Hameed *et al.*, 2014
*Luteibacter*	Rh, Re	Chelates ferric ions, solubilizes monocalcium phosphate, and IAA production.	Guglielmetti *et al.*, 2013
*Demequina*	Rh, Re	Unknown.	—
*Planctomyces*	Rh, Re	Phosphorus removal and oxidation of ammonium. There are no studies on beneficial effects on host plants.	Ma *et al.*, 2018
*Winogradskyella*	Rh, Re	Unknown.	—
*Ilumatobacter*	Rh	Unknown.	—
*Halochromatium*	Rh	Sulfur-oxidizing activity. There are no studies on beneficial effects on host plants.	Montoya *et al.*, 2011
*Candidatus Alysiosphaera*	Rh	Unknown.	—
*Sva0081 sediment group*	Rh	Unknown.	—
*Pir4 lineage*	Rh	Unknown.	—
*Labrenzia*	Re	Unknown. Isolated from the root endosphere of halophytes.	Bibi *et al.*, 2014; Fidalgo *et al.*, 2016
*BD1-7 clade*	Re	Unknown.	—
*Sulfurimonas*	Re	Host detoxification by the oxidation of sulfide.	Fahimipour *et al.*, 2017; Hasler-Sheetal and Holmer, 2015
*Vibrio*	Re	IAA production.	Gutierrez *et al.*, 2009

IAA, indole-3-acetic acid.

**Table 3.** Possible functions of common and abundant bacterial genera in the root endosphere and/or rhizosphere of *Glaux maritima* across Lake Notoro and Lake Tofutsu.

Genus	Sample type	Possible function	References
*Aquibacter*	*Rh, Re*	Zeaxanthin-producing activity. There are no studies on beneficial effects on host plants.	Hameed *et al.*, 2014
*Ilumatobacter*	*Rh, Re*	Unknown.	—
*Pir4 lineage*	*Rh, Re*	Unknown.	—
*Planctomyces*	*Rh*	Phosphorus removal and oxidation of ammonium. There are no studies on beneficial effects on host plants.	Ma *et al.*, 2018
*Winogradskyella*	*Rh*	Unknown.	—
*Blastocatella*	*Rh*	Unknown. One of the abundant genera present in the rice rhizosphere.	Osman *et al.*, 2017
*Sneathiella*	*Rh*	Unknown.	—
*Demequina*	*Rh*	Unknown.	—
*Halioglobus*	*Rh*	Unknown.	—
*Rhodopirellula*	*Rh*	Unknown.	—
*Haliangium*	*Rh*	Anti-fungal metabolite haliangicin production	Fudou *et al.*, 2001
*Paraglaciecola*	*Re*	Unknown.	—
*Photobacterium*	*Re*	Mercury-reducing activity and IAA production.	Mathew *et al.*, 2015
*Marinomonas*	*Re*	N_2_ fixation, phosphate solubilization, IAA production, siderophore production, ACC deaminase activity.	Mesa *et al.*, 2015
*Rhizobium*	*Re*	N_2_ fixation, phosphate solubilization, IAA production, siderophore production, ACC deaminase activity.	Jha *et al.*, 2012; Sorty *et al.*, 2016
*Labrenzia*	*Re*	Unknown. Isolated from the root endosphere of halophytes.	Bibi *et al.*, 2014; Fidalgo *et al.*, 2016
*Vibrio*	*Re*	IAA production.	Gutierrez *et al.*, 2009
*Actinoplanes*	*Re*	IAA, IPYA and GA_3_ productions, anti-fungal activity.	El-Tarabily *et al.*, 2009

IAA, indole-3-acetic acid; ACC, 1-aminocyclopropane-1-carboxylate; IPYA, indole-3-pyruvic acid; GA_3_, gibberellic acid.
