# Supplementary material for: Comparative Analysis of Bacterial Diversity and Community Structure in the Rhizosphere and Root Endosphere of Two Halophytes, Salicornia europaea and Glaux maritima, Collected from Two Brackish Lakes in Japan
Source: Microbes Environ. 2020 Sep 4;35(3):ME20072. doi: 10.1264/jsme2.ME20072 (PMC7511784; doi:10.1264/jsme2.ME20072)

Fig. S1. Rarefaction curves based on the sequences of the V3–V4 region of the 16S rDNA from each sample related to *S. europaea* (A) and *G. maritima* (B) collected from two brackish lakes. Error bars represent the standard error of four replicates. N, Lake Notoro; T, Lake Tofutsu; SE, *S. europaea*; GM, *G. maritima*; Bl, bulk control soil; Rh, rhizosphere; Re, root endosphere.

(A)

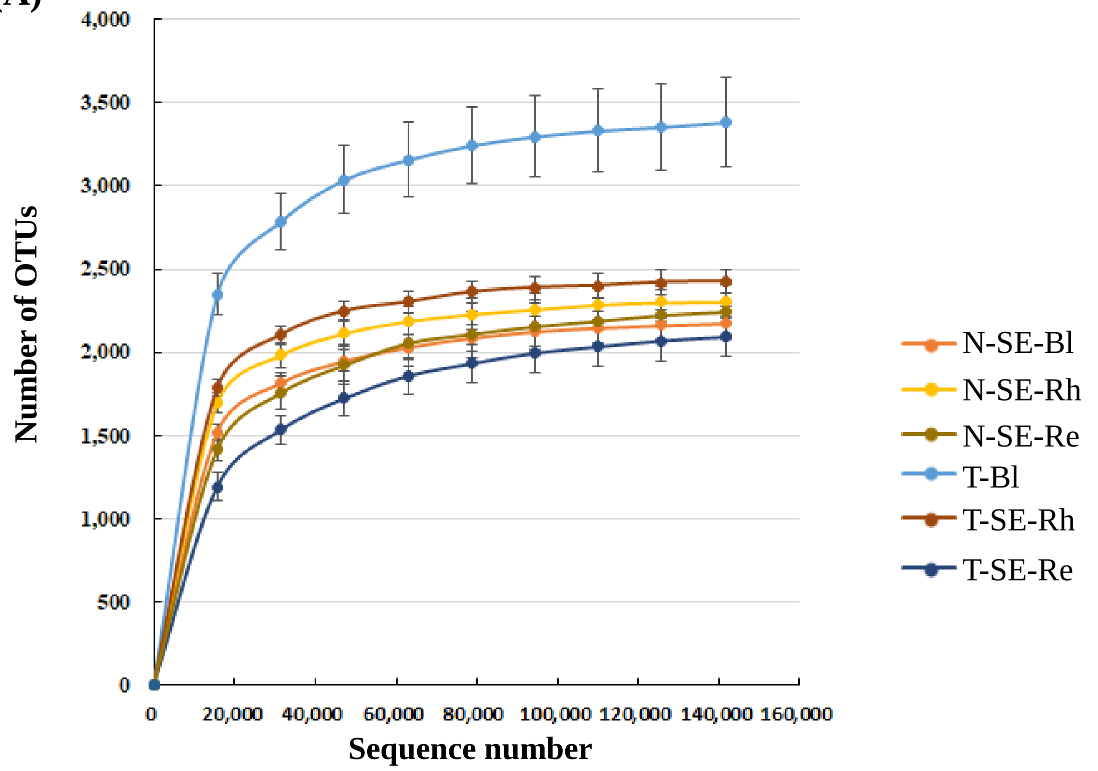

(B)

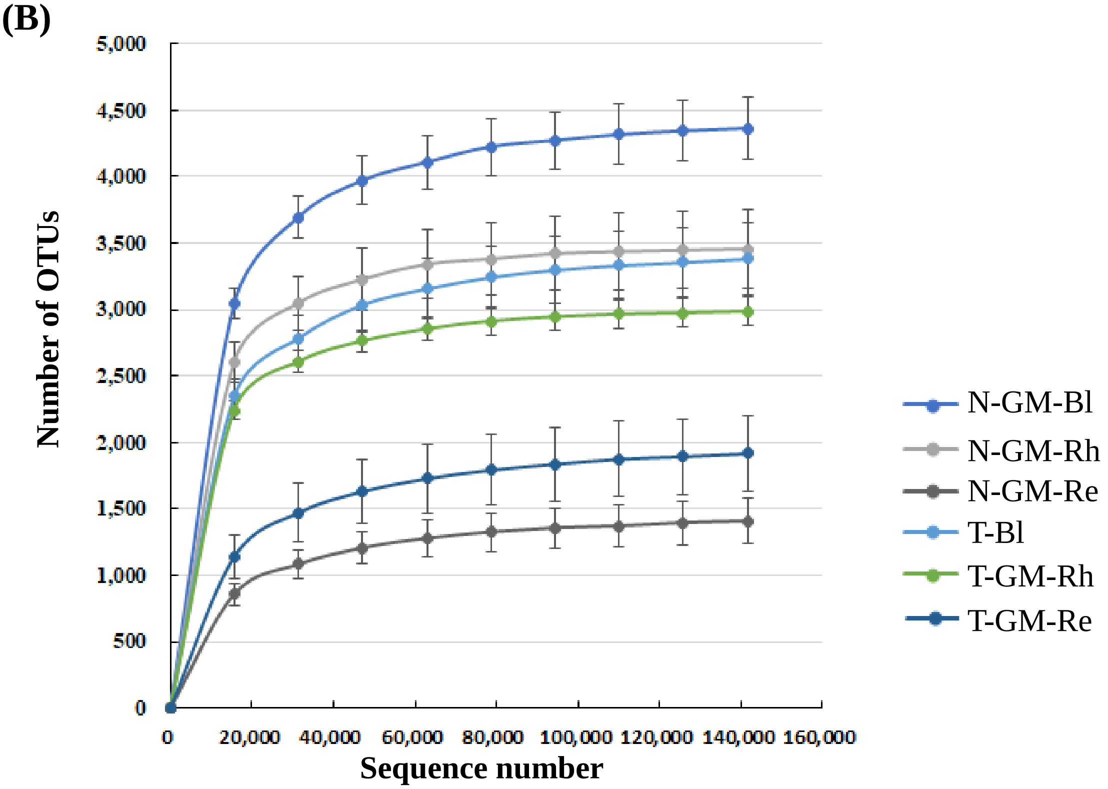

Supplement: Supplementary file 1 — Supplementary Material 1 [file 35_20072_s1.pdf]
